# Stress Preconditioning of Spreading Depression in the Locust CNS

**DOI:** 10.1371/journal.pone.0001366

**Published:** 2007-12-26

**Authors:** Corinne I. Rodgers, Gary A. B. Armstrong, Kelly L. Shoemaker, John D. LaBrie, Christopher D. Moyes, R. Meldrum Robertson

**Affiliations:** Department of Biology, Queen's University, Biosciences Complex, Kingston, Ontario, Canada; Columbia University, United States of America

## Abstract

Cortical spreading depression (CSD) is closely associated with important pathologies including stroke, seizures and migraine. The mechanisms underlying SD in its various forms are still incompletely understood. Here we describe SD-like events in an invertebrate model, the ventilatory central pattern generator (CPG) of locusts. Using K^+^ -sensitive microelectrodes, we measured extracellular K^+^ concentration ([K^+^]_o_) in the metathoracic neuropile of the CPG while monitoring CPG output electromyographically from muscle 161 in the second abdominal segment to investigate the role K^+^ in failure of neural circuit operation induced by various stressors. Failure of ventilation in response to different stressors (hyperthermia, anoxia, ATP depletion, Na^+^/K^+^ ATPase impairment, K^+^ injection) was associated with a disturbance of CNS ion homeostasis that shares the characteristics of CSD and SD-like events in vertebrates. Hyperthermic failure was preconditioned by prior heat shock (3 h, 45°C) and induced-thermotolerance was associated with an increase in the rate of clearance of extracellular K^+^ that was not linked to changes in ATP levels or total Na^+^/K^+^ ATPase activity. Our findings suggest that SD-like events in locusts are adaptive to terminate neural network operation and conserve energy during stress and that they can be preconditioned by experience. We propose that they share mechanisms with CSD in mammals suggesting a common evolutionary origin.

## Introduction

Neural function under stressful conditions is contingent upon maintaining ion gradients across cell membranes, which are essential for neuronal signaling. Hyperthermic failure of neural operation is associated with a loss of ion homeostasis and a major redistribution of ions, including K^+^ [1], [2]. This pattern of ionic disturbance, monitored by an abrupt rise in extracellular potassium ([K^+^]_o_) and depression of electrical activity, shares many characteristics of cortical spreading depression (CSD [3]). In mammalian tissue CSD has been associated with several important pathologies including stroke, seizures and migraine [4]–[6]. In an insect model system, the onset of hyperthermic failure can be preconditioned by prior stress [7]. Preconditioning resulting in ischaemic tolerance is considered an evolutionarily conserved phenomenon of cerebral plasticity, evident in invertebrates and vertebrates including humans [8]. Here we characterize stress-induced neural depression of ventilatory central pattern generation in the migratory locust to determine its similarity to CSD and we tested the role of the Na^+^/K^+^ ATPase in mediating the preconditioning effects of prior heat shock (HS) on hyperthermic neural failure.

Ventilation in locusts is under the control of a reliable central pattern generator (CPG) located in the metathoracic ganglion (MTG) [9], [10] which is sensitive to several forms of stress including changes of internal CO_2_ [11], [12], pH [13] and temperature [14], [15], [12], [7]. In particular, increased temperature elevates the cycle frequency of the ventilatory motor pattern from 1 cycle/s at room temperature to 3 cycles/s just prior to hyperthermic failure at 38–40°C [7]. A subsequent return to room temperature permits recovery of pattern generation within 2 to 3 minutes. Several important neural properties are protected by prior HS (for review, see refs. 2, 16). HS-mediated thermoprotection of the ventilatory CPG results in an increase in the failure temperature and a decrease in the time taken to recover, and can be mimicked by octopamine acting through a cAMP signaling pathway [17]. Given that circulating octopamine increases after heat stress in locusts [18], that octopamine reduces K^+^ conductances and increases activity of the Na^+^/K^+^ ATPase [19] and that HS reduces whole cell K^+^ currents in locust metathoracic neurons [20], we hypothesized that HS preconditioning acts by stabilizing K^+^ homeostasis.

We compared the dynamics of [K^+^]_o_ during failure of motor pattern generation induced by different stressors (hyperthermia, anoxia, ATP depletion, K^+^ injection) and used ouabain (a specific Na^+^/K^+^ ATPase toxin) both to induce repetitive, propagating waves of ionic disturbance and to test the role of the Na^+^/K^+^ ATPase in HS-mediated thermoprotection. We demonstrate that the surges of [K^+^]_o_ associated with cessation of ventilatory motor patterning represent SD-like phenomena whose occurrence can be interpreted as an adaptive response to conserve energy in the locust, rather than the culmination of cellular collapse. Using TTX and TEA we show that SD-like behaviour is not activity-dependent and that some part of the [K^+^]_o_ surge is via TEA-sensitive K^+^ channels. Finally we show that the hyperthermic SD-like event can be preconditioned by a prior HS acting to increase the rate of [K^+^]_o_ clearance, and that the effect of HS is not obviously mediated by the Na^+^/K^+^ ATPase.

## Results

### Stress-induced failure of the ventilatory motor pattern correlates with a sharp rise in extracellular potassium

**Figure 1:**
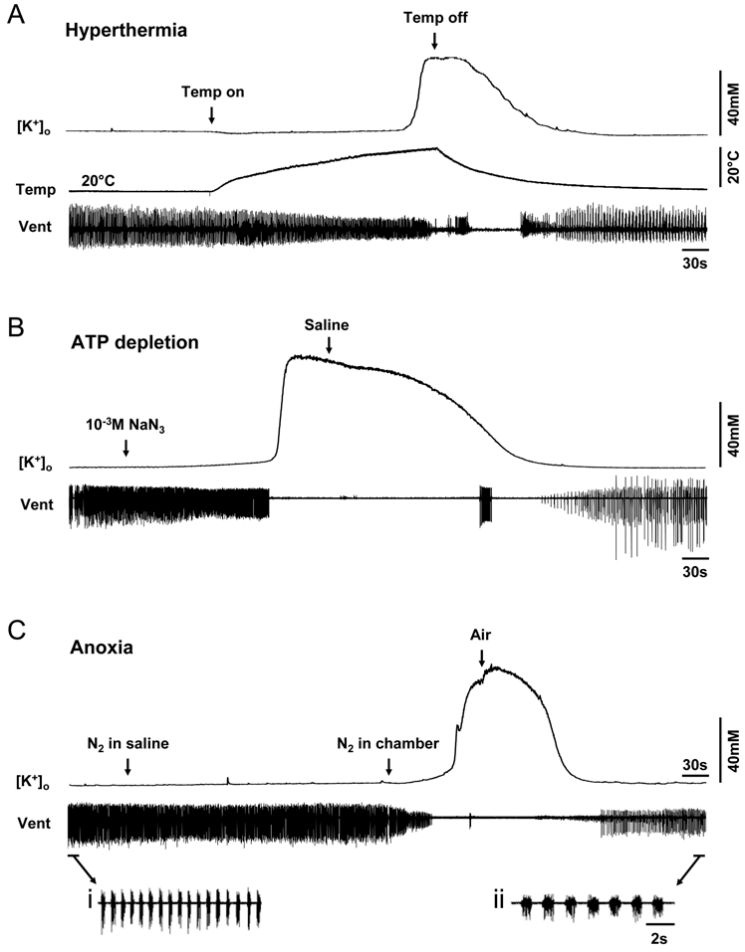
Stress-induced motor pattern failure is associated with surges of [K^+^]_o_. Simultaneous recordings of the ventilatory motor pattern (Vent), the temperature of the superfusing saline at the MTG (Temp in A) and the extracellular potassium concentration ([K^+^]_o_). A. An abrupt increase in [K^+^]_o_ was reliably associated with heat-induced failure of the ventilatory motor pattern, which occurred in 100% of preparations (N = 17). [K^+^]_o_ was restored to normal baseline levels if heat was removed and this was associated with recovery of ventilatory motor patterning. B. 10^−3^ M NaN_3_ was bath-applied until 1 minute post-failure (N = 6). [K^+^]_o_ gradually decreased and the motor pattern recovered upon superfusion of standard locust saline. C. N_2_ was bubbled into the superfusing saline for 5 minutes, then blown over the preparation until 1 minute post-failure (N = 18). Re-oxygenation resulted in [K^+^]_o_ clearance and recovery of the motor pattern. i and ii show expansions of the motor pattern trace pre- and post-stress to show more clearly the ventilatory motor pattern. In B and C the temperature was constant at room temperature (∼22°C).

**Figure 2:**
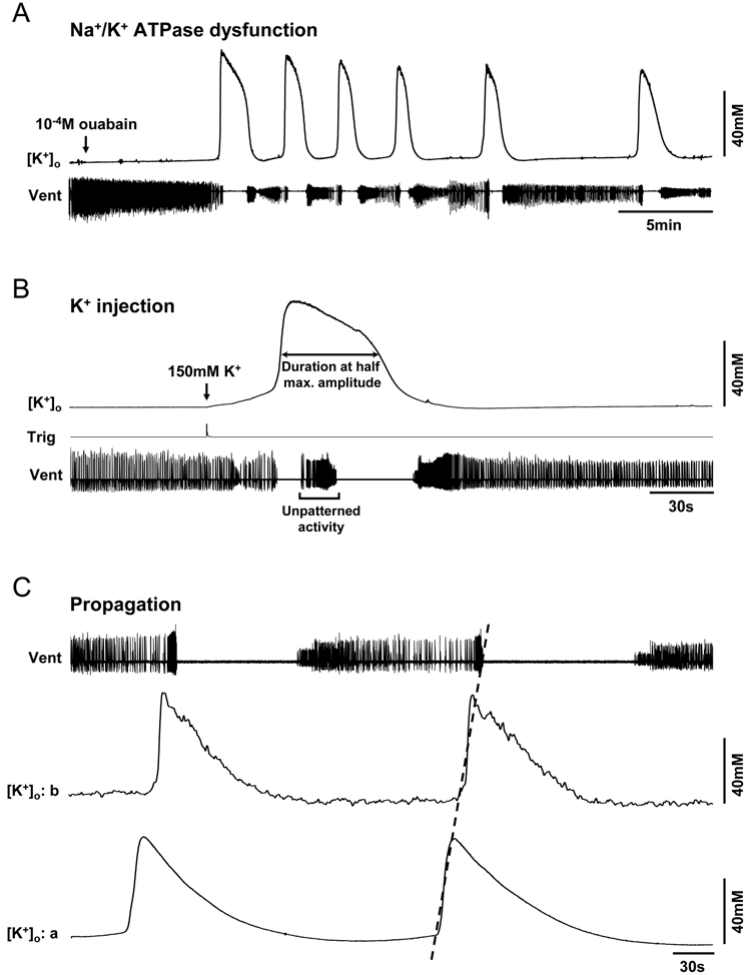
Characteristics of [K^+^]_o_ surges. Simultaneous recordings of the ventilatory motor pattern (Vent), a monitor of pressure-injection of a bolus of K^+^ within the MTG (Trig in B) and the extracellular potassium concentration ([K^+^]_o_). A. Continuous bath application of 10^−4^ M ouabain elicited multiple surges in [K^+^]_o_ (N = 13). In the experiment shown here it took 434 seconds for 10^−4^ M ouabain to penetrate the MTG and induce failure of motor pattern generation. B. A 35 nl pressure-injection of locust saline containing a 15-fold higher [K^+^] (150 mM compared to 10 mM) into the MTG neuropile was sufficient to bring [K^+^]_o_ to threshold and induce an abrupt surge (N = 18). C. Two K^+^ -sensitive microelectrodes were inserted in different regions of the MTG (a and b) to illustrate propagation (dotted line). In this experiment the propagation speed was 1.9 mm/min.

Ventilatory motor pattern activity and extracellular potassium ion concentrations in the MTG were measured during treatment with various cellular stressors in order to establish a relationship between stress-induced circuit failure and an extracellular build-up of potassium ions (**Figures 1** and **2**). Hyperthermic failure of the ventilatory CPG was reliably associated with an abrupt increase in [K^+^]_o_, which occurred in 100% of CON preparations (**Figure 1A**) and of all preparations irrespective of preconditioning or pharmacological treatment (N_total_ = 85). When the temperature was allowed to return to baseline levels, [K^+^]_o_ remained elevated before gradually decreasing (**Figure 1A**). Post-stress recovery of the ventilatory central pattern generator correlated with restoration of [K^+^]_o_ to initial levels (**Figure 1A**). In addition, abrupt surges in [K^+^]_o_ were reliably triggered by ATP depletion using 10^−3^ M sodium azide (NaN_3_) and anoxia (100% N_2_) (**Figure 1B and C**). Peak [K^+^]_o_ reached during surges induced by hyperthermia (52±2 mM), NaN_3_ (85±2 mM) and anoxia (79±3 mM) were different (one-way ANOVA, *P*<0.001, *F*_(2,38)_ = 43.634). The peak [K^+^]_o_ induced by hyperthermia was lower than the [K^+^]_o_ peaks induced by NaN_3_ and anoxia (*post hoc* Tukey tests, *P*<0.05). The recovered motor pattern was robust but not identical to the pre-stress motor pattern (see e.g. **Figure 1C** **i** and **ii**).

Abrupt surges in [K^+^]_o_ were reliably triggered by Na^+^/K^+^ ATPase inhibition using 10^−4^ M ouabain and by locally increased [K^+^]_o_ within the ganglion (**Figure 2**). Continuous bath application of 10^−4^ M ouabain elicited multiple surges in [K^+^]_o_ where the rise and fall of [K^+^]_o_ was associated with failure and recovery of the ventilatory motor pattern, respectively (**Figure 2A**). The time from ouabain application to the midpoint of the initial [K^+^]_o_ increase was 557±43 seconds. The average [K^+^]_o_ surge period from the first to the second surge was 217±16 seconds. [K^+^]_o_ increased to a mean peak of 63±5 mM during the initial surge induced by 10^−4^ M ouabain and subsequently returned to a mean baseline level of 10±0.2 mM. [K^+^]_o_ clearance always coincided with recovery of motor pattern generation, however the ventilatory rhythm frequency and duration became more variable following each [K^+^]_o_ surge (**Figure** **2A**). A 35 nl injection of locust saline containing a 15-fold higher [K^+^] (150 mM compared to 10 mM) was sufficient to bring [K^+^]_o_ to threshold for induction of an abrupt surge (**Figures 2B** and S**1**). The peak [K^+^]_o_ reached during surges induced by K^+^ injection was 63±8 mM. The average duration of the [K^+^]_o_ surge, measured at half the maximum amplitude, was 64±9 seconds. It was of interest to determine if the repetitive ionic disturbance is localized to one area in the MTG, or if, similar to CSD, there is propagation across different areas of the locust nervous system. To investigate this we measured [K^+^]_o_ simultaneously at two different locations in the MTG ∼0.4 to 0.6 mm apart (Figure 2C). Abrupt surges in [K^+^]_o_ generated by injection of high [K^+^] saline into the extracellular space spread locally to other areas of the MTG at a speed of 2.4±0.04 mm/min (**Figure 2C**), but did not propagate through the connectives to the mesothoracic ganglion and vice versa (N = 3; data not shown).

### The hyperthermic [K^+^]_o_ event does not represent complete collapse of the K^+^ gradient

**Figure 3:**
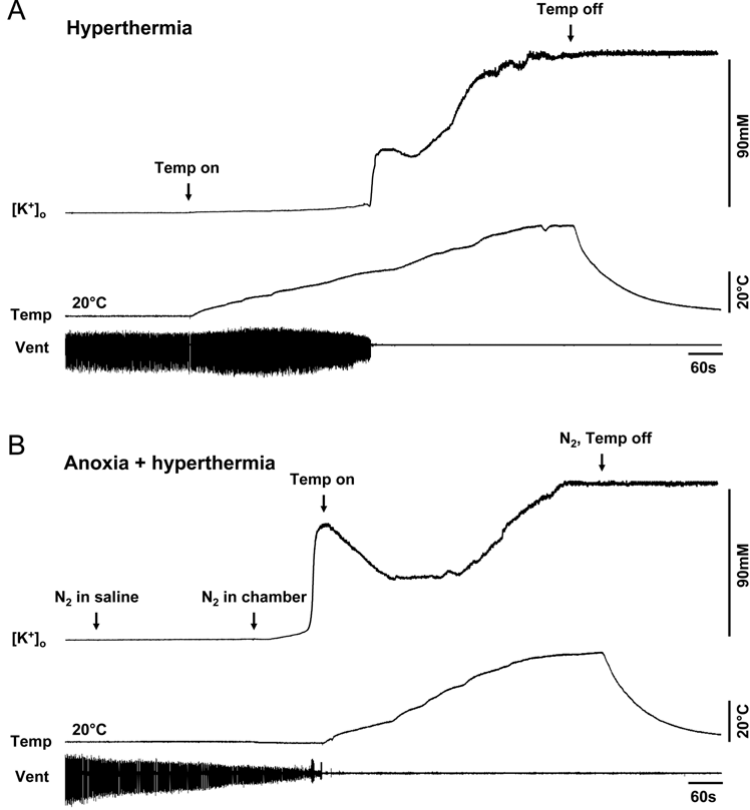
The hyperthermic [K^+^]_o_ event is not representative of complete collapse of the K^+^ gradient. A. During a continuous increase of temperature [K^+^]_o_ increased sharply at ∼40°C coincident with motor pattern failure and continued to rise until a second plateau was reached at ∼60°C. When the heater was turned off [K^+^]_o_ remained elevated and motor pattern generation failed to recover (N = 11). B. Only the second plateau was evident during temperature increase to ∼60°C following prior anoxic arrest of motor pattern generation. When the N_2_ was turned off and internal temperature returned to room temperature [K^+^]_o_ remained elevated and motor pattern generation failed to recover (N = 12).

To determine if the peak [K^+^]_o_ represented complete collapse of the [K^+^]_o_ gradient during hyperthermia, temperature was increased beyond failure of the motor pattern until a second [K^+^]_o_ plateau was reached at ∼60°C (100±7 mM; **Figure 3A**). The motor pattern did not recover upon return to room temperature and [K^+^]_o_ remained elevated. Hyperthermia following anoxic arrest of the motor pattern eliminated the hyperthermic [K^+^]_o_ event and only the second plateau (113±6 mM) was evident without recovery (**Figure 3B**) suggesting that these different stressors converge on the same tissue response. There was no difference in the ultimate [K^+^]_o_ at ∼60°C between the two treatment groups (hyperthermia alone and anoxia-induced failure of the motor pattern prior to hyperthermia) (*t*-test, *t = *−1.370, *P = *0.185, d.f. = 21).

### The hyperthermic [K^+^]_o_ event is delayed by Na^+^ channel block and diminished by K^+^ channel block

**Figure 4:**
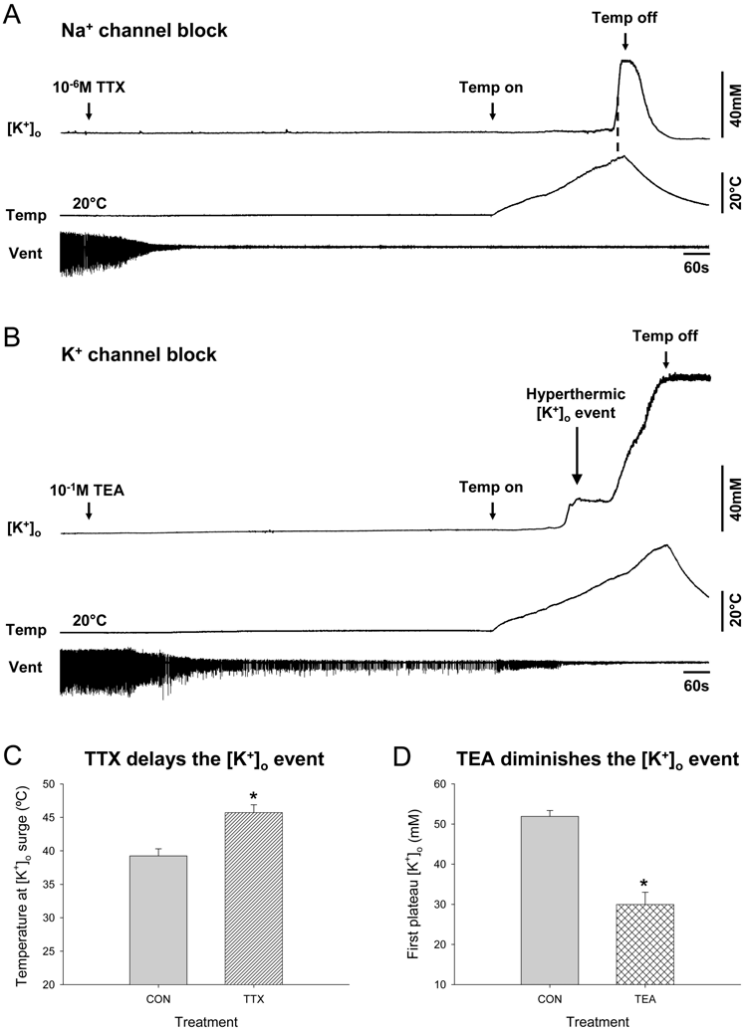
The hyperthermic [K^+^]_o_ event is delayed by blocking Na^+^ channels and neural activity using TTX and is diminished by blocking K^+^ channels using TEA. A. 10^−6^ M TTX abolished electrical activity within minutes while [K^+^]_o_ remained stable. The abrupt rise in [K^+^]_o_ was not significantly diminished by TTX. The hyperthermic [K^+^]_o_ event occurred at a significantly higher temperature (measured at dotted line) in the absence of electrical activity (46±1°C) compared to CON locusts (39±1°C) (C) (N_CON_ = 17; N_TTX_ = 15). B. 10^−1^ M TEA significantly reduced the hyperthermic [K^+^]_o_ event (N_CON_ = 17; N_TEA_ = 6) (D). [K^+^]_o_ increased with continued temperature increase to ∼60°C.

We used voltage-gated Na^+^ and K^+^ channel blockers (TTX and TEA, respectively) to determine if the hyperthermic SD-like event in the locust is dependent on neural activity and if K^+^ efflux is at least in part via TEA-sensitive K^+^ channels. Bath application of 10^−6^ M TTX quickly abolished electrical activity from ventilatory muscle 161 while [K^+^]_o_ remained stable at ∼10 mM for 15 minutes prior to the temperature ramp (**Figure 4A**). However, occurrence of the hyperthermic [K^+^]_o_ event was TTX-resistant; there was no difference in the peak [K^+^]_o_ level between CON and TTX locusts (52±2 mM and 50±4 mM respectively; *t*-test, *t = *0.334, *P = *0.741, d.f. = 30). The abrupt rise in [K^+^]_o_ was delayed in TTX-treated locusts, occurring at higher temperatures during the continuous ramp increase than in CON locusts (46±1°C versus 39±1°C; *t*-test, *t = *−4.541, *P<*0.001, d.f. = 30) (**Figure 4C**). Due to the effectiveness of the sheath enclosing the contents of the MTG, bath application of a high concentration of TEA (10^−1^ M) was required to successfully abolish motor pattern generation, although electrical activity was still detected from muscle 161. The hyperthermic [K^+^]_o_ event occurred at 38.9±0.6°C in locusts treated with 10^−1^ M TEA compared to 39.3±0.9°C (no significant difference) in CON locusts (**Figure 4B**) but was reduced in amplitude (30±3 mM versus 52±2 mM; *t*-test, *t = *5.429, *P<*0.001, d.f. = 21) (**Figure 4D**). With continued temperature increase to ∼60°C [K^+^]_o_ reached a higher second plateau in TEA-treated locusts (109±9 mM) that was not different from the second plateau in CON locusts (*t*-test, *t = *−0.755, *P = *0.462, d.f. = 15).

### Initiation of [K^+^]_o_ surges are not correlated with ATP levels within the ganglion

**Figure 5:**
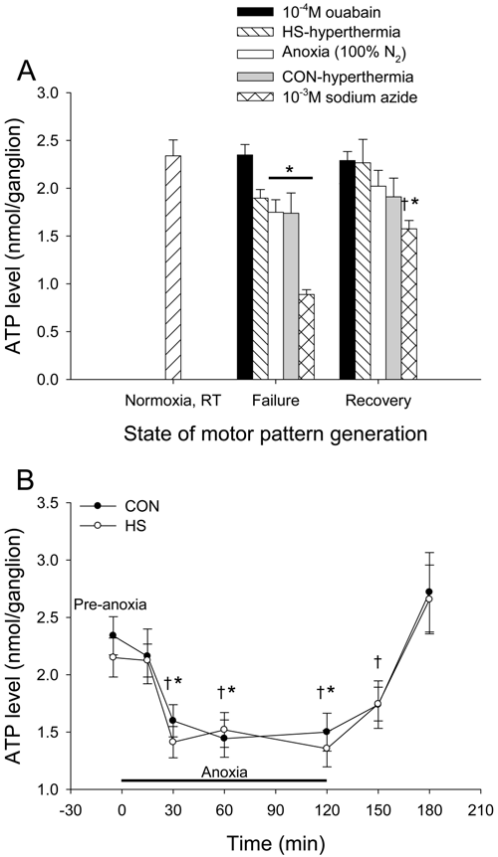
Failure and recovery of the CPG are not dependent on MTG ATP and HS preconditioning does not confer protection by improving ATP availability. A. ATP levels did not differ from the pre-stress level (Normoxia at room temperature; N = 16) in locusts treated with 10^−4^ M ouabain or hyperthermia (HS) at failure or recovery, or in all other groups at recovery following stress-induced failure with the exception of NaN_3_-treated locusts. There was a main effect of treatment driven by significant differences among groups within failure and recovery (not indicated by symbols). There was no effect of HS pre-treatment on ATP levels at failure or recovery of motor pattern generation. Asterisks indicate significant differences from the pre-stress level and daggers indicate significantly different ATP levels at failure and recovery in ganglia of NaN_3_-treated locusts. Sample sizes (failure, recovery): N_Ouabain_ = 10,11; N_HS-hyperthermia_ = 8,8; N_Anoxia_ = 9,8; N_CON-hyperthermia_ = 7,7; N_Sodium azide_ = 8,9. B. CON and HS locusts did not have significantly different ATP levels during anoxia-induced coma or subsequent recovery. ATP levels dropped in both CON and HS locusts after 30 minutes of anoxia and remained stable until locusts were removed from the anoxic environment. ATP levels increased to around pre-anoxia values after one hour in normally oxygenated air. Sample sizes (CON, HS): N_0min_ = 16,16; N_15min_ = 12,11; N_30min_ = 11,11; N_60min_ = 9,9; N_120min_ = 12,11; N_150min_ = 11,12; N_180min_ = 10,10. Asterisks indicate significant differences from 0 and 15 minutes and daggers indicate significant differences from 180 minutes (or 60 minutes recovery) according to *post hoc* Tukey tests (*P*<0.05).

Cell energetics may play a role in the initiation of, and recovery from, the [K^+^]_o_ event induced by various cellular stressors. ATP levels in the MTG taken from locusts subjected to hyperthermia before and after heat shock preconditioning (CON and HS), 10^−4^ M ouabain, 10^−3^ M sodium azide, and anoxia (100% N_2_) were compared prior to stress, at failure and at subsequent recovery of the motor pattern (**Figure 5A**). Ganglionic ATP levels were depressed at failure induced by anoxia, hyperthermia (CON), and NaN_3_ and at subsequent recovery in NaN_3_-treated locusts (*post hoc* Dunnett's test, *P*<0.05). Stress treatment and the state of motor pattern generation, i.e. failure vs. recovery, both affected ATP levels (two-way ANOVAs: *P*<0.001, *F*_(4,75)_ = 17.435; *P* = 0.002, *F*_(1,75)_ = 10.193). At failure of the motor pattern, NaN_3_-treated locusts had lower ganglionic ATP levels than all other groups. Locusts subjected to anoxia and hyperthermia (CON) had lower ATP levels than locusts treated with 10^−4^ M ouabain (*post hoc* Tukey tests, *P*<0.05). At recovery of the motor pattern NaN_3_-treated locusts had lower ganglionic ATP levels than locusts subjected to 10^−4^ M ouabain and hyperthermia (HS) suggesting that recovery was independent of metabolic state (*post hoc* Tukey tests, *P*<0.05). ATP levels in ganglia at failure and recovery of the motor pattern were not different except in NaN_3_-treated locusts (*post hoc* Tukey tests, *P*<0.05). Notably, HS preconditioning did not increase ATP levels at hyperthermic failure or subsequent recovery of the motor pattern compared to CON-hyperthermia locusts (**Figure 5A**). The lack of effect of HS on MTG ATP levels was also evident during and after two hours of whole animal anoxia (**Figure 5B**). HS pre-treatment did not affect ATP levels prior to anoxia, at 15, 30, 60, and 120 minutes of anoxia exposure, and at 30 and 60 minutes post-anoxia (two-way ANOVA, *P* = 0.467, *F*_(1,147)_ = 0.531). Anoxia exposure affected ganglionic ATP (two-way ANOVA, *P*<0.001, *F*_(6,147)_ = 11.418). Anoxia (15 minutes) did not affect ATP levels in either CON or HS locusts, even though locusts entered anoxic coma within 1 minute. ATP levels dropped after 30 minutes of anoxia and remained stable for 90 minutes in CON and HS locusts (*post hoc* Tukey tests, *P*<0.05). Following 2 hours in an anoxic environment, ATP increased after 60 minutes of normoxia in CON and HS locusts (*post hoc* Tukey tests, *P*<0.05).

### HS pre-treatment changed [K^+^]_o_ homeostasis in the MTG

**Figure 6:**
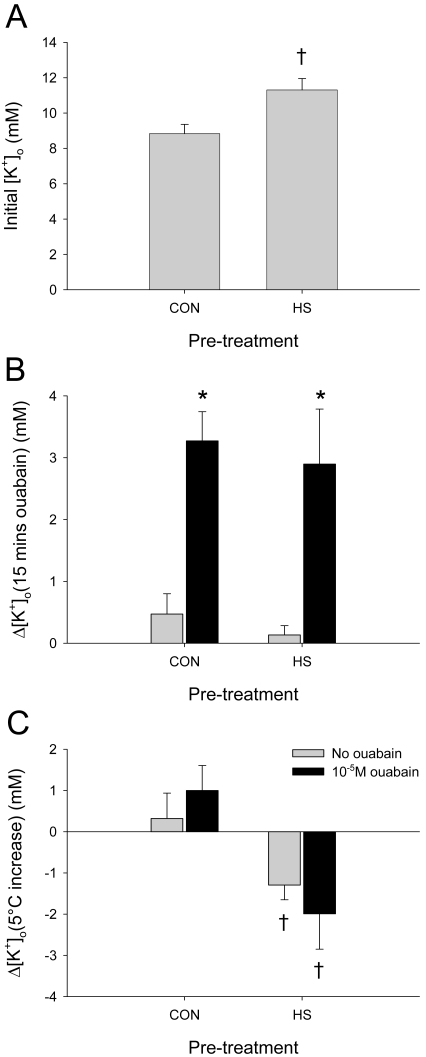
HS and ouabain affect [K^+^]_o_. A. Heat shock preconditioning resulted in a significantly higher [K^+^]_o_ in HS locusts compared to CON locusts. All animals pooled: N_CON_ = 25; N_HS_ = 26. B. CON-OUA and HS-OUA locusts had a significantly greater increase in [K^+^]_o_ after 15 minutes of bath application with 10^−5^ M ouabain compared to CON and HS locusts. HS pre-treatment did not have an effect on Δ[K^+^]_o_ over this 15 minute period (N_CON_ = 12; N_CON-OUA_ = 10; N_HS_ = 17; N_HS-OUA_ = 8). C. CON and HS locusts had a markedly different Δ[K^+^]_o_ in response to a 5°C increase in temperature. Δ[K^+^]_o_ was positive in CON locusts and negative in HS locusts after only one minute of temperature increase. There was no effect of 10^−5^ M ouabain treatment on Δ[K^+^]_o_ one minute into the temperature ramp (N_CON_ = 12; N_CON-OUA_ = 10; N_HS_ = 17; N_HS-OUA_ = 8). Asterisks indicate a significant effect of ouabain and daggers indicate a significant effect of pre-treatment according to *t*-test (A) and *post hoc* Tukey tests (*P*<0.05) (B,C).

We used 10^−5^ M ouabain to test whether increased Na^+^/K^+^ ATPase activity could underlie the increased thermotolerance conferred by HS pre-treatment [7]. 10^−5^ M ouabain is not normally sufficient to induce the SD-like events of **Figure 2A** but is sufficient to inhibit the Na^+^/K^+^ ATPase (see below). HS pre-treatment and 10^−5^ M ouabain bath application affected [K^+^]_o_ before and during a temperature ramp (**Figure 6**). Heat shock pre-treatment increased initial [K^+^]_o_ measured after 10 minutes of bath application with standard locust saline (*t*-test, *t = *−3.195, *P = *0.002, d.f. = 49; **Figure 6A**). Changes in extracellular potassium (Δ[K^+^]_o_) were examined after 15 minutes of ouabain treatment at room temperature and in response to the initial 5°C increase of the temperature ramp to determine the effects of HS pre-treatment and 10^−5^ M ouabain treatment on K^+^ homeostasis (**Figure 6B and C**). HS pre-treatment had no effect on Δ[K^+^]_o_ after 15 minutes of bath application, however there was an effect of 10^−5^ M ouabain treatment (two-way ANOVAs: *P* = 0.407, *F*_(1,43)_ = 0.703; *P*<0.001, *F*_(1,43)_ = 42.772) (**Figure 6B**). CON-OUA and HS-OUA locusts had a greater increase in [K^+^]_o_ than CON and HS locusts after 10^−5^ M ouabain bath application for 15 minutes (*post hoc* Tukey tests, *P*<0.05). HS pre-treatment affected Δ[K^+^]_o_ after a 5°C increase in temperature, however there was no effect of 10^−5^ M ouabain treatment (two-way ANOVAs: *P*<0.001, *F*_(1,43)_ = 15.584; *P* = 0.988, *F*_(1,43)_ = 0.000223) (**Figure 6C**). CON and CON-OUA locusts had a greater Δ[K^+^]_o_ than HS and HS-OUA locusts (*post hoc* Tukey tests, *P*<0.05).

### Time to recovery is correlated with failure temperature in control and HS locusts

**Figure 7:**
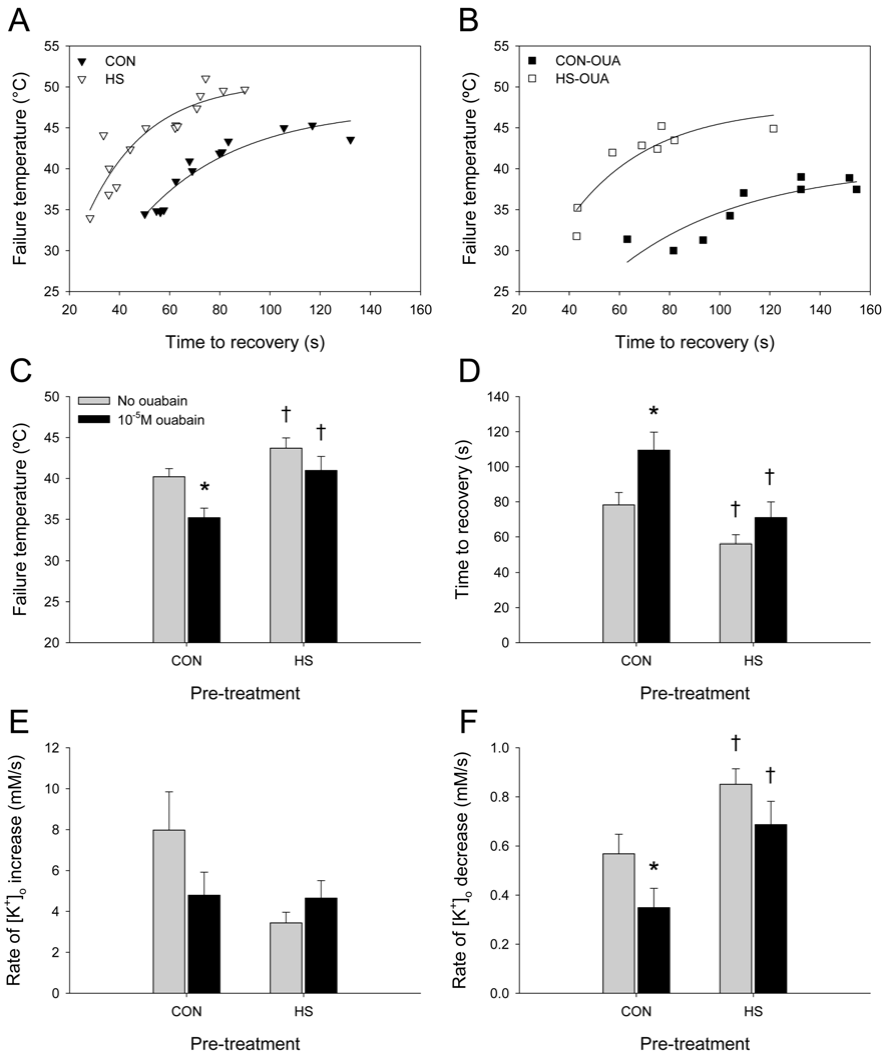
HS delays the [K^+^]_o_ event and speeds recovery by increasing the rate of [K^+^]_o_ clearance. A. Time to recovery was positively correlated with failure temperature in CON and HS locusts (N_CON_ = 13; N_HS_ = 15). Animals whose ventilatory motor pattern failed at a higher temperature had a longer time to recovery when temperature returned to normal levels. B. The correlations seen in CON and HS animals were shifted in animals treated with 10^−5^ M ouabain (N_CON-OUA = _9; N_HS-OUA = _8). The relationship between failure temperature and time to recovery in HS-OUA animals resembled CON animals. Data points for each group were fitted with exponential curves that rise to a plateau (A, B). C. Ventilatory motor pattern generation in HS locusts failed at a significantly higher temperature than in CON locusts with and without 10^−5^ M ouabain treatment. There was a significant effect of ouabain on the ability to withstand high temperature stress in CON locusts but not in HS locusts (N_CON_ = 15; N_CON-OUA_ = 9; N_HS_ = 17; N_HS-OUA_ = 8). D. Ventilatory motor pattern generation in HS locusts took significantly less time to recover following heat-induced failure than in CON locusts with and without ouabain treatment. CON-OUA locusts took significantly more time to recover than CON locusts, however there was no significant effect of ouabain treatment on time to recovery in HS locusts (N_CON_ = 13; N_CON-OUA_ = 10; N_HS_ = 15; N_HS-OUA_ = 8). E. Overall there were no main effects of HS pre-treatment or ouabain treatment on the rate of [K^+^]_o_ increase (mM/s) associated with failure of the motor pattern (N_CON_ = 15; N_CON-OUA_ = 10; N_HS_ = 18; N_HS-OUA_ = 7). F. The rate of [K^+^]_o_ decrease (mM/s) associated with recovery of the motor pattern was significantly greater in HS locusts than in CON locusts and this effect of pre-treatment was conserved in ouabain-treated locusts. Ouabain treatment significantly decreased the rate of [K^+^]_o_ clearance in CON locusts but not in HS locusts (N_CON_ = 15; N_CON-OUA_ = 10; N_HS_ = 18; N_HS-OUA_ = 8). Asterisks indicate a significant effect of ouabain and daggers indicate a significant effect of pre-treatment according to *post hoc* Tukey tests (*P*<0.05) (C–F).

HS preconditioning delayed the onset of the [K^+^]_o_ event and subsequently hastened recovery and re-establishment of normal [K^+^]_o_ (**Figure 7**). However HS had no effect on the amplitude of the [K^+^]_o_ event at failure (**Figure S2**) and there was no correlation between time to recovery and peak [K^+^]_o_ in C and HS locusts (data not shown) indicating that the effect of HS on rate of recovery was not due to a decrease in the initial ionic disturbance. There was a strong positive correlation between time to recovery and failure temperature in control locusts that was shifted by HS and by 10^−5^ M ouabain (Pearson Product Moment Correlations: CON, *r* = 0.91, *P*<0.0001; HS, *r* = 0.90, *P*<0.0001; CON-OUA, *r* = 0.88, *P* = 0.00172; HS-OUA, *r* = 0.85, *P* = 0.00785) (**Figure 7A and B**). Thus high failure temperatures were correlated with slower recovery under all conditions. HS improved performance by increasing failure temperatures and speeding recovery whereas ouabain impaired performance of both CON and HS preparations.

### HS pre-treatment increases failure temperature and decreases time to recovery

HS pre-treatment increased the upper temperature limit and shortened time to recovery when these measures were examined independently (**Figure 7C and D**). HS pre-treatment and 10^−5^ M ouabain treatment affected failure temperature though there was no interaction between treatments suggesting that the effect of ouabain did not depend on the pre-treatment (two-way ANOVAs: *P* = 0.001, *F*_(1,45)_ = 11.777; *P* = 0.007, *F*_(1,45)_ = 8.140) (**Figure 7C**). HS increased failure temperatures compared with CON, and ouabain decreased failure temperatures in CON but not HS preparations (*post hoc* Tukey tests, *P*<0.05). HS pre-treatment and 10^−5^ M ouabain treatment also affected time to recovery of the motor pattern with a similar lack of interaction (two-way ANOVAs: *P*<0.001, *F*_(1,42)_ = 15.302; *P* = 0.005, *F*_(1,42)_ = 8.823) (**Figure 7D**). HS decreased time to recovery compared with CON, and ouabain increased time to recovery in CON but not HS preparations (*post hoc* Tukey tests, *P*<0.05).

### HS pre-treatment increases the rate of [K^+^]_o_ clearance following hyperthermic failure

Rates of [K^+^]_o_ increase and clearance associated with hyperthermic failure and subsequent recovery of the ventilatory motor pattern were compared among groups to determine if preconditioning improves [K^+^]_o_ stabilization and to test the role of the Na^+^/K^+^ ATPase in this preconditioning (**Figure 7E and F**). Neither HS pre-treatment nor 10^−5^ M ouabain treatment affected the rate of [K^+^]_o_ increase associated with failure of the motor pattern (two-way ANOVAs: *P* = 0.098, *F*_(1,46)_ = 2.846; *P* = 0.482, *F*_(1,46)_ = 0.502) (**Figure 7E**). However HS pre-treatment and 10^−5^ M ouabain treatment did affect the rate of [K^+^]_o_ decrease associated with recovery of the motor pattern (two-way ANOVAs: *P*<0.001, *F*_(1,47)_ = 14.395; *P* = 0.024, *F*_(1,47)_ = 5.478) (**Figure 7F**) though there was no interaction between the treatments. HS increased the [K^+^]_o_ clearance rate compared with CON, and ouabain decreased this measure in CON but not in HS preparations (*post hoc* Tukey tests, *P*<0.05). Thus the proportion of the [K^+^]_o_ clearance rate sensitive to the 10^−5^ M ouabain treatment was halved by HS from ∼40% in control preparations to ∼20% after HS.

### HS preconditioning does not increase Na^+^/K^+^ ATPase activity in the MTG

To complement the results obtained by ouabain treatment on [K^+^]_o_ clearance rates we also measured steady-state Na^+^/K^+^ ATPase activity within the MTG. Na^+^/K^+^ ATPase activity (i.e. the ouabain-sensitive fraction of total MTG ATPase activity) was 144±12 nmol ATP/min/mg protein in CON ganglia compared to 133±9 nmol ATP/min/mg protein in HS ganglia (no significant difference; *t*-test, *t = *0.755, *P = *0.468, d.f. = 10; N_CON_ = 6, N_HS_ = 6). In the presence of phosphatase inhibitors, Na^+^/K^+^ ATPase activity was 120±7 nmol ATP/min/mg protein in CON ganglia compared to 116±10 nmol ATP/min/mg protein in HS ganglia (no significant difference; *t*-test, *t = *0.430, *P = *0.672, d.f. = 18; N_CON_ = 10, N_HS_ = 10). This suggests that total Na^+^/K^+^ ATPase activity is not a target for protection by HS preconditioning.

## Discussion

In a central pattern generator controlling ventilation in the locust, we have shown that hyperthermic failure and recovery of neural function are tightly correlated with an abrupt rise in [K^+^]_o_ and its subsequent restoration when temperature is allowed to return to normal. Anoxia and ATP depletion evoked similar tissue responses, which could also be induced when we disturbed the ionic equilibrium by impairing Na^+^/K^+^ ATPase activity using ouabain or by local neuropile injections of high [K^+^] saline. The fact that a prior anoxia-induced [K^+^]_o_ surge occluded the hyperthermic event suggests that these different stressors converged on the same mechanism. In addition, the nature of these events as large ionic disturbances propagating throughout the MTG neuropile makes it unlikely that different stressors would have additive effects. The amplitude of the hyperthermic [K^+^]_o_ surge was smaller than those induced by either anoxia or ATP depletion and we attribute this partly to the confounding effect of its occurrence at high temperatures (>40°C). This is supported by further analysis that shows a significant negative correlation between the failure temperature during hyperthermia (for CON, HS, CON-OUA, HS-OUA preparations) and [K^+^]_o_ at its peak (Pearson Product Moment Correlation; *r* = −0.3, *P* = 0.03; N = 47, one outlier removed), and may be due to the fact that at the point of hyperthermic failure [K^+^]_o_ increased from its room temperature level (*t*-test, *t* = −5.085, *P*<0.001, d.f. = 104) thereby reducing the driving force on K^+^ ions. We conclude that the different stressors converged on a single tissue mechanism.

CSD occurs in vertebrate grey matter and is characterized by a rapid and nearly complete depolarization of neurons that propagates at 2–5 mm/min as a wave across the cortex. This propagating wave is due to a disordered regulation of ionic homeostasis evident as a massive redistribution of ions across neuronal membranes, including large increases of [K^+^]_o_ [4]. Each wave results in neuronal hyperexcitation followed by suppression of electrical activity because of spike inactivation. SD events can be evoked by hypoxia [1], [4], [21], ouabain [22], [1], [4], simulated ischemia [23], and increased [K^+^]_o_ [1], [4], [21] with normal temperature, as well as hyperthermia [1]. The locust responses: 1) were all-or-none events of similar magnitude (at least with respect to [K^+^]_o_), 2) were induced by the same stimuli, 3) were not activity-dependent, 4) occurred as repetitive waves with ouabain, 5) propagated at around 2 mm/min, 6) did not cross between ganglia through the connectives (equivalent to white matter) and 7) were associated with complete depression of neural activity manifest by failure of ventilatory pattern generation. Thus the locust tissue response, reflected in the all-or-none surge of [K^+^]_o_, shares most, if not all, of the essential characteristics of CSD.

The biophysical mechanism of the extensive redistribution of ions causing CSD is incompletely understood. It nevertheless seems clear that SD-like events in mammals are triggered by positive feedback processes involving an initial ionic disturbance reaching a threshold and caused by neuronal overexcitation or by treatments or conditions that impair ionic homeostasis (e.g. refs. 24, 25, 22, 4). In a model hippocampal pyramidal neuron, SD-like events can be triggered by manipulating ion clearance mechanisms or levels of excitation, causing the net current in the apical dendrites to turn inward [21]. The locust responses were triggered by treatments that cause a reduction in energy (i.e. ATP depletion and anoxia). However disruption of energy status was not essential since similar events were triggered by impairment of the Na^+^/K^+^ ATPase (10^−4^ M ouabain) and by local injection of high [K^+^] saline into the neuropile. Moreover there was no correlation between the occurrence of motor pattern failure and recovery with levels of ATP in the MTG. Energy status of a cell (e.g. phosphorylation potential) can change and activate signaling pathways without measureable effects on ATP [26], [27], but the size of the sample precluded measurement of any adenylate but ATP. Hyperthermia caused a drop in metathoracic ATP but also increased levels of neuronal excitation in the ganglion. There was an interesting trade-off in our measures of thermotolerance for ventilatory pattern generation such that early failure (i.e. low failure temperature) was associated with shorter times to recovery. This suggests that the triggered event is adaptive and represents a neural off-switch to conserve energy and to prevent hyperexcitation during stressful conditions. In contrast to the insect system, CSD in mammals is associated with local tissue hypoxia and neuronal swelling due to energy consumption that is not met with adequate oxygen supply [28]. The recovery of motor function was closely associated with the return of [K^+^]_o_ to near normal levels and thus the time taken to recover was related to the rate of clearance of extracellular K^+^ ions. Treatment with 10^−5^ M ouabain rendered preparations more thermosensitive by decreasing failure temperature and increasing recovery time concomitant with a decrease in the rate of extracellular K^+^ ion clearance during the recovery period. This indicates an important role for the Na^+^/K^+^ ATPase pump, as in vertebrate SD-like phenomena. The [K^+^]_o_ disturbance induced by injection of high [K^+^] saline propagated within the locust MTG at a similar rate as the depolarizing wave in mammalian brain slices during CSD. Studies suggest opening of intercellular gap junctions as a mechanism of SD propagation in vertebrates [29], [30]. Similar mechanisms may exist in the insect CNS in which glia are involved in ion homeostasis, function, and development of the insect brain [31].

Our findings show that the events we recorded in locust ganglia are SD-like ionic disturbances similar to CSD and possibly induced by common mechanisms, which in theory would suggest that these mechanisms have been evolutionarily conserved. Moreover in the locust there is evidence that this is an adaptive event to conserve energy and prevent complete cellular collapse. In the case of hyperthermia this was evident when temperature was increased to the point at which [K^+^]_o_ levels reached a high plateau and motor pattern recovery was precluded.

We found that prior HS preconditioned neural function in the metathoracic ganglion, allowing the ventilatory pattern generator to operate at higher temperatures and to recover more rapidly from hyperthermic failure on return to normal temperatures. Such preconditioning significantly protects neural function and has been well established in insect preparations [2], [16]. HS signals a change to more extreme environmental conditions and it has pleiotropic effects to prepare cells, tissues and organisms for such a change. The shift to higher temperatures of the neural off-switch described here would be appropriate for an organism that has put protective mechanisms in place with a robust HS response [32], [33]. Here we show that at least part of the beneficial effect of HS was on the rate of clearance of extracellular K^+^ ions with no change in the initial disturbance. There was no evidence that this was due to improvements of energy status or management; ATP levels in the MTG after HS were not different from control either at rest, during anoxic stress or during hyperthermic stress. Given the thermoprotective effect of a heat shock protein (Hsp70) on mammalian muscle sarco/endoplasmic reticulum Ca^2+^ ATPase [34] we suspected that the SD-like event in the locusts might have been shortened by a protective effect of HS on the activity of the Na^+^/K^+^ ATPase pump. However measurement of ouabain-sensitive ATPase activity in a biochemical assay of the metathoracic ganglion was not different after HS, with or without the presence of phosphatase inhibitors. Measurements of Na^+^/K^+^ ATPase activity were made on ganglia of CON and HS locusts at rest, and it is possible that examination of pump activity when stimulated by a rise in [K^+^]_o _would yield different results suggesting a role for the pump. In addition, this result does not rule out the possibility that HS affected the trafficking of Na^+^/K^+^ ATPase complexes in neuronal and glial membranes. Nevertheless an effective concentration of ouabain (10^−5^ M) did not prevent the protective effects of HS on K^+^ clearance in the MTG. Indeed the ouabain-sensitive proportion of extracellular K^+^ ion clearance was reduced by HS suggesting either that HS somehow rendered membrane-bound Na^+^/K^+^ ATPase less sensitive to ouabain or that HS was acting via a different mechanism such as glial buffering via inwardly rectifying K^+^ channels. This issue is currently unresolved.

In summary we conclude that in locust CNS different stressors converge on an adaptive SD-like event that can be preconditioned by prior HS to delay its occurrence until higher temperatures. We believe that the mechanisms underlying this event are much like those underlying CSD and other SD-like phenomena in mammals and a conserved genetic component to this thermotolerance pathway in insects has recently been demonstrated [35]. It is therefore an intriguing possibility that this phenomenon in the locust and migraine in humans [36]–[38] have common evolutionary origins.

## Materials and Methods

### Animals

Adult male locusts, *Locusta migratoria migratorioides* (R. and F.), 4–6 weeks past the imaginal ecdysis, were randomly chosen from a crowded colony maintained in the Department of Biology at Queen's University. The animals were housed in cages within the colony under a 12 h:12 h light:dark cycle. Each cage was individually lit with a 40 W fluorescent light bulb. Room temperature was maintained at 25±1°C, with a constant humidity of 23±1%. Animals were provided with wheat seedlings and carrot slices once daily and an *ad libitum* mixture of 1 part skim milk powder, 1 part torula yeast, and 13 parts bran, by volume. All animals were collected from the colony at the same time each morning and were placed in a two litre ventilated plastic container for 4–8 hours prior to experimentation.

### Heat shock pre-treatment

Locusts designated for control (CON) and HS pre-treatments were separated into different containers. HS locusts were placed in a humid incubator (45°C) for 3 hours followed by 1–5 hours of recovery at room temperature (21±2°C). CON animals were held at room temperature for 4–8 hours.

### Preparation of potassium-sensitive microelectrodes

[K^+^]_o_ was measured with K^+^ -sensitive microelectrodes. The K^+^ -sensitive microelectrodes were made from unfilamented glass pipettes (0.1 mm diameter; World Precision Instruments Inc., Sarasota, FL, USA) that were cleaned with methanol (99.9%) and dried on a hot plate, then pulled to form a low resistance (5–7 MΩ) tip. The microelectrodes were silanized by exposure to dichlorodimethylsilane (99%) (Sigma-Aldrich) vapor while baking on a hot plate (100°C) for one hour. After cooling, the microelectrodes were filled at the tip with Potassium Ionophore I-Cocktail B (5% Valinomycin; Sigma-Aldrich) to form an artificial membrane and then back-filled with 500 mM KCl. The tips of the K^+^ -sensitive microelectrodes were suspended in distilled water until experimentation. Reference electrodes were made by pulling a filamented pipette (0.1 mm diameter; World Precision Instruments Inc.) to form a low resistance (5–7 MΩ) tip and were filled with 3 M KCl.

### Semi-intact preparation

Following removal of legs, wings and pronotum, the nervous system and ventilatory muscle 161 were exposed by making a dorsal midline incision and pinning the locust open onto a cork board, dorsal side up. The gut, air sacs and fat bodies were removed. A Peri-Star peristaltic pump (World Precision Instruments Inc.) was used for perfusion of standard locust saline into the thoracic and abdominal cavities which contained (in mM) 147 NaCl, 10 KCl, 4 CaCl_2_, 3 NaOH, and 10 HEPES buffer (pH 7.2) (all chemicals were from Sigma-Aldrich). The saline flow was directed onto the MTG where the ventilatory CPG is located and exited through an incision in the posterior abdominal wall. The tissue covering the MTG was removed as well as the cuticle and attached muscle tissue situated between the connectives. A metal plate was inserted below the MTG to stabilize it. Nerves five and seven of the MTG were cut on both sides to allow saline and pharmacological agents to permeate the ganglion. The preparation was grounded by placing a silver wire in the posterior tip of the abdomen.

### Electromyographic recording of the motor pattern

An extracellular recording of the ventilatory motor pattern was obtained by placing a 0.1 mm diameter copper wire, insulated except at the tip, onto abdominal muscle 161. The recording was digitized using a DigiData 1200 Series Interface (Axon Instruments Inc., Union City, CA, USA) and displayed using AxoScope 9.0 software (Axon Instruments Inc.). Ventilation can be monitored as a series of rhythmic electrical bursts in the abdominal muscles (e.g. Fig. 1d and e). Failure was characterized as the cessation of electrical activity in the extracellular recording and the lack of visible contractions of the ventilatory muscles. Recovery was defined as the first visible sign of rhythmical abdominal contractions coincident with resumption of rhythmic electrical activity.

### Extracellular potassium recording

K^+^ -sensitive microelectrodes were connected to a pH/Ion amplifier (Model 2000; A-M Systems Inc., Carlsborg, WA, USA) by an electrode holder with a chloride-coated silver wire. Preceding each experiment, a K^+^ -sensitive and a reference electrode were calibrated at room temperature (∼21°C) using 15 mM and 150 mM KCl solutions to determine the voltage change, or ‘slope’, which was needed for determination of [K^+^]_o_ (mM) using the Nernst equation. Electrode sensitivities ranged from 54 to 58 mV for a 10-fold change in [K^+^]. A reference and K^+^ -sensitive electrode pair were discarded if the slope did not fall within this range or if the voltage reading exhibited atypical sensitivities. The K^+^ -sensitive microelectrode was inserted through the sheath of the MTG adjacent to the reference electrode in the area of the ventilatory CPG.

The voltage of the K^+^ -sensitive electrode is logarithmically related to the potassium concentration which was obtained by transforming the [K^+^]_o_ trace in mV to mM using the Nernst equation:
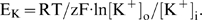
In the above equation, E_K_ is the membrane potential at which K^+^ is at equilibrium, R is the gas constant (8.315 J · K^−1^ · mol^−1^), T is the temperature in ° Kelvin (T_Kelvin_ = 273.16 + T_Celsius_), F is Faraday's constant (96,485 C · mol^−1^), z is the valence of the ion, and [K^+^]_o_ and [K^+^]_i_ are the concentrations of K^+^ outside and inside the cell, respectively. For a membrane permeable only to K^+^ at room temperature (20°C), substituting the appropriate numbers and converting natural log (ln) into log base 10 (log_10_) results in the equation:
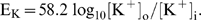
The K^+^-sensitive electrode voltage recording was set to zero in a stock solution of 15mM KCl and the following conversion of the above equation was used for determination of extracellular potassium concentrations (corrections for temperature differences were made when appropriate):
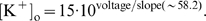



### Hyperthermia

Preparations were bathed for 25 minutes with saline before starting the temperature ramp to allow the ventilatory rhythm to stabilize and the [K^+^]_o_ to settle at around 10mM. Saline passed through a glass pipette wrapped in Nichrome^TM^ wire, and the temperature of the saline was controlled by varying the amount of current passed through the wire. Temperature at the ganglion was monitored using a thermocouple connected to a digital thermometer (BAT-12; Physitemp Instruments Inc., Clifton, NJ, USA). Internal temperature was raised in a ramped manner 5°C per minute from room temperature (∼20°C) until failure of the motor pattern (**Figure 1A**, **Figure 6C**, and **Figure 7**), until the abrupt rise in [K^+^]_o_ was observed (**Figure 4A**), or raised beyond the first [K^+^]_o_ plateau (not necessarily in a ramped manner) until a second plateau was reached (**Figure 3** and **Figure 4B**). At this time, the heater was switched off allowing the saline temperature to return to ambient levels and the motor pattern to recover.

### Anoxia

The locust preparation was enclosed in a sealed chamber. Nitrogen gas (N_2_) was first bubbled into the superfusing saline for 5 minutes then blown over the preparation within the chamber. 100% N_2_ was maintained until 1 minute post-failure of the motor pattern at which point the preparation was re-oxygenated.

### Pharmacological treatments

All drugs were dissolved in standard locust saline and bath-applied in semi-intact preparations as described above (all drugs were obtained from Sigma-Aldrich). Preparations were bathed in standard locust saline for a 10 minute stabilization period prior to pharmacological treatment. Drugs were bath-applied continuously for the full duration of each experiment with the exception of 10^−3^ M sodium azide (NaN_3_), an electron transport chain inhibitor that prevents cells from using oxygen, which was bath-applied until 1 minute post-failure. 10^−4^ M ouabain, a Na^+^/K^+^ ATPase toxin, was bath-applied continuously for 1 hour. 10^−6^ M tetrodotoxin (TTX), a Na^+^ channel blocker, and 10^−1^ M tetraethylammonium chloride (TEA), a voltage-gated K^+^ channel blocker, were bath-applied for 15 minutes prior to a ramped increase in temperature (5°C per minute). For TTX experiments, the heater was turned off once the [K^+^]_o_ surge was observed and the temperature at the [K^+^]_o_ surge was taken as the temperature at half the maximum amplitude of the [K^+^]_o_ increase. For TEA experiments, the temperature ramp was continued beyond the first [K^+^]_o_ plateau until a second plateau was reached, at which point the heater was turned off.

CON and HS locusts were treated with 10^−5^ M ouabain to determine the role of ouabain-sensitive Na^+^/K^+^ ATPase activity in thermotolerance and [K^+^]_o_ homeostasis. Animals were divided into four groups: **1)** CON, N = 17; **2)** HS, N = 18; **3)** CON locusts treated with 10^−5^ M ouabain (CON-OUA), N = 10; **4)** HS locusts treated with 10^−5^ M ouabain (HS-OUA), N = 8. Experiments on animals receiving different (pre-) treatments were interspersed over time with each other. All preparations were bathed for 25 minutes with saline before starting the temperature ramp. For 10^−5^ M ouabain treatment, the ganglion was bathed with standard locust saline for 10 minutes, and then with 10^−5^ M ouabain for 15 minutes prior to the temperature ramp. At 25 minutes, internal temperature was raised in a ramped manner 5°C per minute until failure of the motor pattern. Failure temperature (°C) and time to recovery (s) were scored as the temperature at which rhythmical ventilator activity ceased and the length of time taken to recover motor pattern function, respectively. In order to determine the nature of the relationships between time to recovery and failure temperature for each group, values were log-transformed to make the relationships linear, which allowed accurate statistical analyses of the data. The correlations are shown in Fig. 7 as scatterplots of the untransformed data fit with exponential curves that rise to a plateau. Rates of [K^+^]_o_ increase and decrease associated with hyperthermic failure and recovery of the motor pattern were also determined. Rate of [K^+^]_o_ increase (mM/s) was calculated as the slope between the baseline at the inflection point of the [K^+^]_o_ increase and the maximum peak [K^+^]_o_. Rate of [K^+^]_o_ decrease (mM/s) was calculated as the slope between the maximum peak [K^+^]_o_ and the baseline [K^+^]_o_ inflection point. The sample sizes for each analysis varied depending on the ability to measure parameters in some or all locusts within each group.

To locally increase [K^+^] in the MTG neuropile we pressure-injected using a PicoSpritzer III (INTRACEL, Shepreth, UK) a small volume (35 nl) of locust saline containing a 15-fold higher [K^+^] (147 mM NaCl, 150 mM KCl, 4 mM CaCl_2_, 3 mM NaOH, 10 mM HEPES buffer, pH 7.2). Two K^+^ -sensitive microelectrodes were inserted into the MTG, one next to the injection point and another in a different region of the ganglion. The distance between the recording electrodes (∼0.4 to 0.6 mm) was determined as well as the time to onset of the [K^+^]_o_ surge measured by each electrode. Using these values the speed of propagation (mm/s) of the [K^+^]_o_ disturbance between the two recording electrodes was calculated.

### ATP extraction and assay for locust ganglia

Metathoracic ganglia were removed from locust preparations prior to stress, at stress-induced failure, and at recovery of the motor pattern for ATP measurement. ATP levels in the ventilatory neuropile of HS locusts at hyperthermic failure and subsequent recovery of the motor pattern were also measured in order to determine if preconditioning confers protection by increasing energy availability. In addition, metathoracic ganglia were removed from CON and HS locusts at normal oxygen levels (pre-anoxia) and at 15, 30, 60, and 120 minutes of anoxia exposure. Locusts were then removed from the anoxic environment and ganglia were removed at 30 and 60 minutes post-anoxia for ATP measurement.

Immediately after removal, each ganglion was added to 220 µl of ice-cold 3.5% perchloric acid in a 1.5 ml centrifuge tube. The ganglion was homogenized thoroughly with a pestle and the solution was centrifuged for 5 minutes at maximum speed. Then, 200 µl of the supernatant were transferred to a new centrifuge tube on ice containing 20 µl Tris (saturated), 20 µl KCl (2 M), 10 µl phenol red (0.005%), and 120 µl KOH (1 M) to neutralize the sample. The neutralized sample was stored at −80°C until the ATP assays were performed. At that time, samples were thawed and centrifuged for five minutes at maximum speed. A volume of 2.5 µl of supernatant was used for the ATP assay.

ATP was quantified using a luciferin-luciferase reaction in which ATP is consumed and light is emitted when firefly luciferase catalyzes the oxidation of D-luciferin. Since ATP is the limiting reagent, the light emitted is proportional to the ATP present, measured by an L_max_ luminometer (Molecular Devices, Sunnyvale, CA, USA). A reaction mix was prepared by diluting one volume of luciferin-luciferase ATP Assay Mix (lyophilized powder containing luciferin, luciferase, MgSO_4_, DTT, EDTA Na_4_, BSA, and tricine buffer salts dissolved in 5 ml of sterile water) with 100 volumes of ATP Assay Mix Dilution Buffer (250 ml: 300 mg MgSO_4_, 4 mg DTT, 100 mg EDTA Na_4_, 250 mg BSA, 2.25 g tricine buffer salts, pH 7.5). The diluted luciferin-luciferase reaction mix was added by auto injector in 200 µl aliquots to 2.5 µl of sample, pre-dispensed in a 96-well microplate. Light was collected for 20 seconds. Measures of light intensity were compared to a standard curve generated using known quantities of ATP prepared in water, acidified and neutralized as were samples.

### Assay of Na^+^/K^+^ ATPase activity in locust MTG

The Na^+^/K^+^ ATPase activity was assessed using a pyruvate kinase/lactate dehydrogenase assay in the presence or absence of ouabain. Single metathoracic ganglia from control and HS locusts were homogenized on ice in 300 µL of lysis buffer (150 mM sucrose, 10 mM EDTA, 50 mM imidazole and 0.1% deoxycholate, pH 7.3). In one experiment, inhibitors of phosphorylation and dephosphorylation were also added to the lysis buffer to prevent changes in the phosphorylation status of the ATPase. The additional compounds included (in mM) 10 EGTA, 50 NaF, 10 tetrasodium pyrophosphate, 1 phospho-serine, 1 phospho-threonine, and 1 phospho-tyrosine. After homogenization, 0.01 volumes of 100 mM PMSF (dissolved in isopropanol) were added to the homogenate.

The measurement of ATPase activity was measured as the production of NADH spectrophotometrically for 10 minutes at 340 nm. Each sample was assayed in six wells on the 96 well plate, three containing ouabain. Each well contained a 300 µL reaction mixture consisting of the following solutions (in order): 228 µL of assay buffer (50 mM imidazole, 100 mM NaCl, 20 mM KCl, and 5 mM MgCl_2_), 3 µL each of 0.2 mM NADH, 0.5 mM PEP, 10 U/mL LDH, and 10 U/mL PK, 15 µL of either ouabain (0.5 mM) or assay buffer, and 30 µL sample homogenate. The reaction was started with the addition of 15 µL ATP (3 mM). The slope of absorbance over 10 minutes was compared between samples with and without ouabain to give a value for activity of only Na^+^/K^+^ ATPase. This value was normalized to total protein within the sample, which was measured from each homogenate using a standard protocol based on the Bradford method [39].

### Statistical analyses

Data were plotted using SigmaPlot 8.0 (SPSS Inc., Chicago, IL, USA) and results are reported as the mean±standard error (s.e.m.). Data were analyzed using SigmaStat 3.0 statistical analysis software (SPSS Inc.) and statistical differences were determined using appropriate parametric tests as indicated in the text. A 95% confidence interval was used to determine significance among means.

## Supporting Information

Figure S1 Gradual increases in the volume of K^+^ injected triggered a tissue response. Simultaneous recordings of the ventilatory motor pattern (Vent), pressure-injection of a bolus of K^+^ within the MTG (Trig) and the extracellular potassium concentration ([K^+^]_o_). Injection of very small volumes of high K^+^ saline triggered a tissue response, however ignition of the all-or-none [K^+^]_o_ event occurred only when a threshold was reached.
(0.89 MB TIF)

Figure S2 The degree of the [K^+^]_o_ disturbance was the same in CON and HS locusts. There were no main effects of HS pre-treatment or 10^−5^ M ouabain treatment on the peak [K^+^]_o_ associated with failure of the motor pattern (N_CON_ = 17; N_CON-OUA_ = 10; N_HS_ = 18; N_HS-OUA_ = 8).
(0.76 MB TIF)
