# Supplementary figures and images for: Stress Preconditioning of Spreading Depression in the Locust CNS
Source: PLoS One. 2007 Dec 26;2(12):e1366. doi: 10.1371/journal.pone.0001366 (PMC2137934; doi:10.1371/journal.pone.0001366)

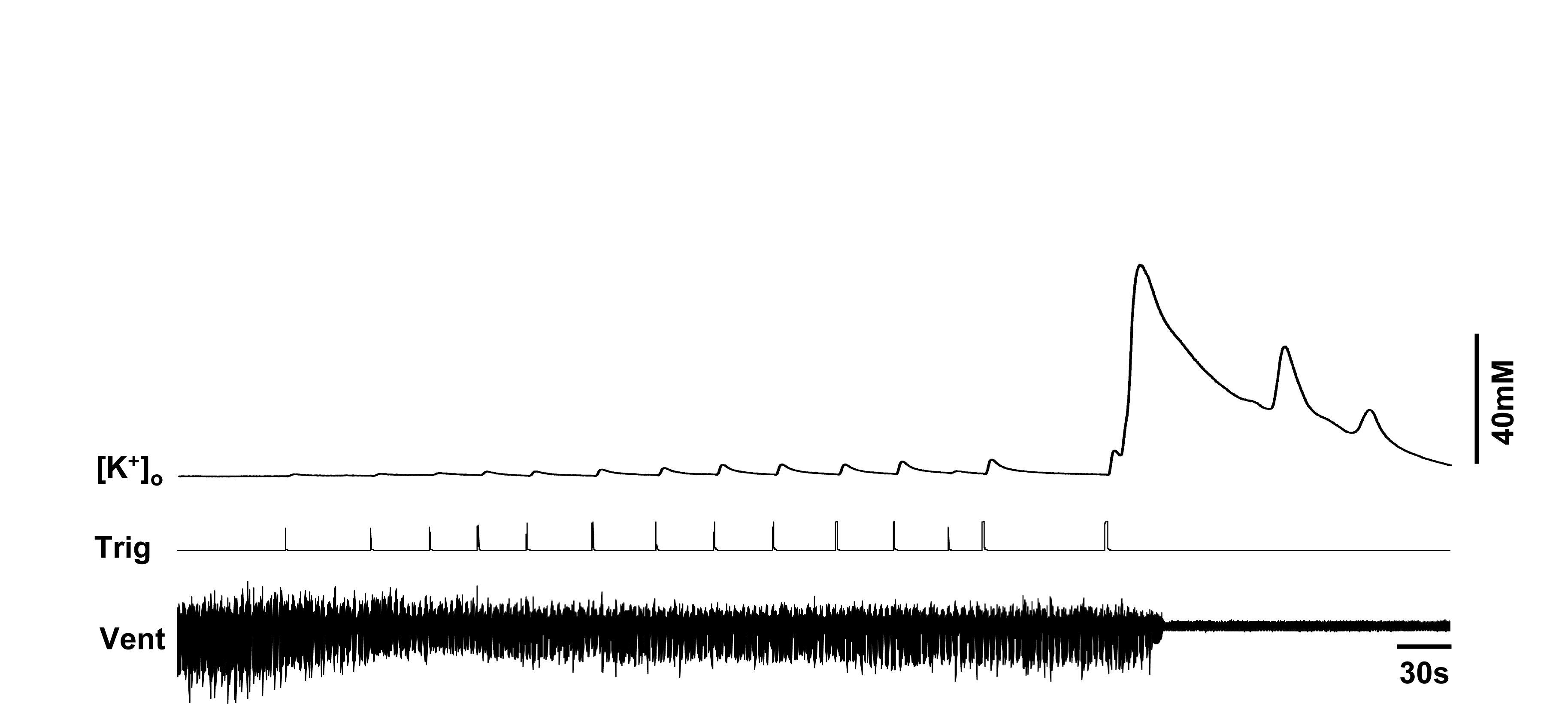

Supplement: Figure S1 — Gradual increases in the volume of K+ injected triggered a tissue response. Simultaneous recordings of the ventilatory motor pattern (Vent), pressure-injection of a bolus of K+ within the MTG (Trig) and the extracellular potassium concentration ([K+]o). Injection of very small volumes of high K+ saline triggered a tissue response, however ignition of the all-or-none [K+]o event occurred only when a threshold was reached. (0.89 MB TIF) [file pone.0001366.s001.tif]

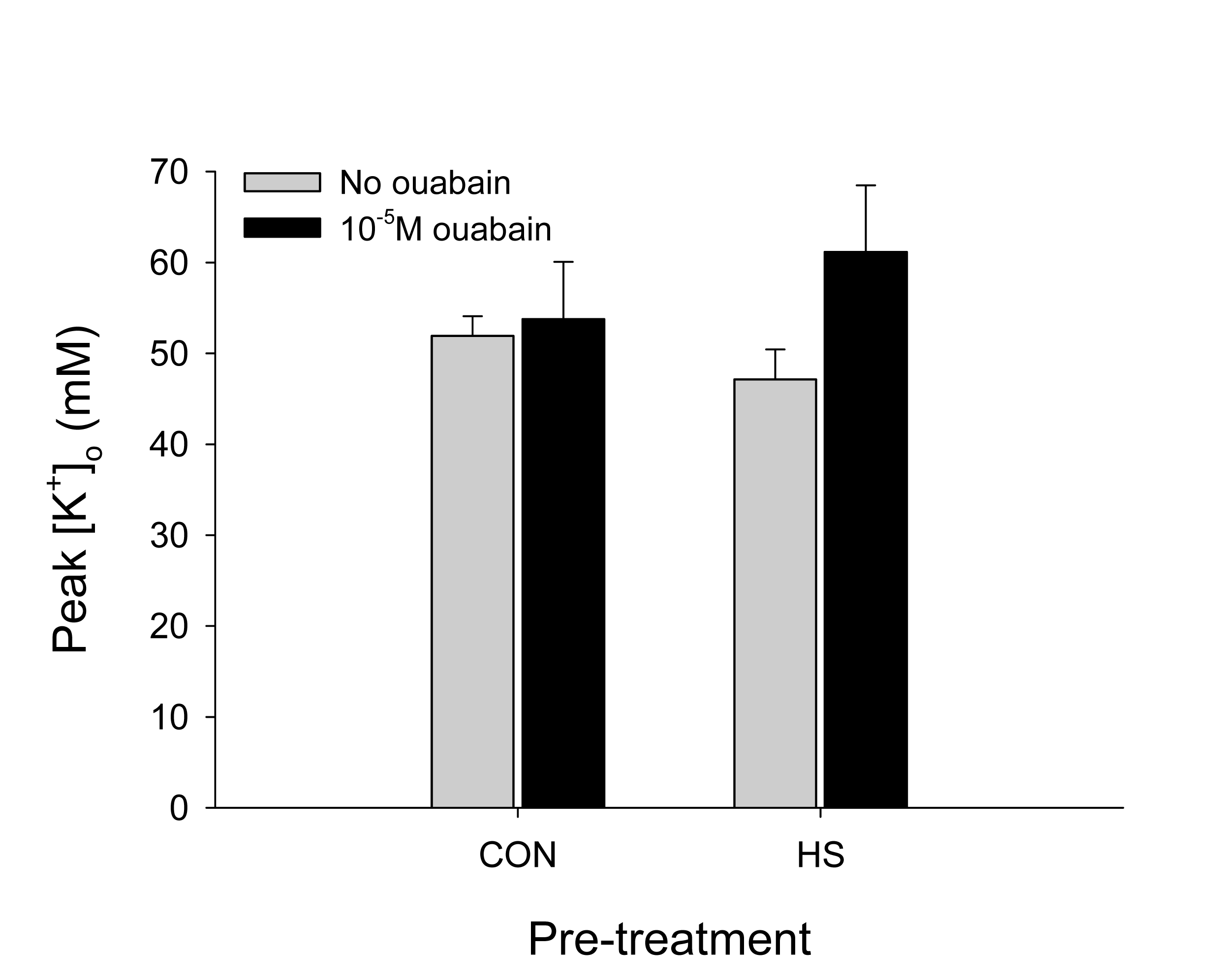

Supplement: Figure S2 — The degree of the [K+]o disturbance was the same in CON and HS locusts. There were no main effects of HS pre-treatment or 10−5 M ouabain treatment on the peak [K+]o associated with failure of the motor pattern (NCON = 17; NCON-OUA = 10; NHS = 18; NHS-OUA = 8). (0.76 MB TIF) [file pone.0001366.s002.tif]
